# Sleep disturbance and anxiety symptoms among asymptomatic COVID-19 carriers in Shanghai, China: the mediating role of entrapment and defeat

**DOI:** 10.1186/s12889-023-15803-8

**Published:** 2023-05-29

**Authors:** Yujie Liu, Xin Ge, Jinxin Zhang, Lulu Xu, Fan Hu, Suping Wang, Jialin Liu, Xiaodong Yang, Dake Shi, Yong Cai

**Affiliations:** 1 Hongqiao International Institute of Medicine, Tongren Hospital, Shanghai Jiao Tong University School of Medicine, Shanghai, 200335 China; 2 School of Public Health, Shanghai Jiao Tong University School of Medicine, Shanghai, 200025 China; 3 Department of Critical Care Medicine, Ruijin Hospital, Shanghai Jiao Tong University School of Medicine, Shanghai, 200025 China; 4 Department of Neurology and Institute of Neurology, Ruijin Hospital, Shanghai Jiao Tong University School of Medicine, Shanghai, 200025 China; 5 Department of Infection Control, Ruijin Hospital, Shanghai Jiao Tong University School of Medicine, Shanghai, 200025 China

**Keywords:** Anxiety symptoms, Sleep disturbance, Entrapment, Defeat, Asymptomatic COVID-19 carrier

## Abstract

**Background:**

The COVID-19 pandemic increases the risk of psychological problems, especially for the infected population. Sleep disturbance and feelings of defeat and entrapment are well-documented risk factors of anxiety symptoms. Exploring the psychological mechanism of the development of anxiety symptoms is essential for effective prevention. This study aimed to examine the mediating effects of entrapment and defeat in the association between sleep disturbance and anxiety symptoms among asymptomatic COVID-19 carriers in Shanghai, China.

**Methods:**

A cross-sectional study was conducted from March to April, 2022. Participants were 1,283 asymptomatic COVID-19 carriers enrolled from the Ruijin Jiahe Fangcang Shelter Hospital, Shanghai (59.6% male; mean age = 39.6 years). Questionnaire measures of sleep disturbance, entrapment, defeat, anxiety symptoms, and background characteristics were obtained. A mediation model was constructed to test the mediating effects of entrapment and defeat in the association between sleep disturbance and anxiety symptoms.

**Results:**

The prevalence rates of sleep disturbance and anxiety symptoms were 34.3% and 18.8%. Sleep disturbance was positively associated with anxiety symptoms (*OR [95%CI]* = 5.013 [3.721–6.753]). The relationship between sleep disturbance and anxiety symptoms (total effect: Std. Estimate = 0.509) was partially mediated by entrapment (indirect effect: Std. Estimate = 0.129) and defeat (indirect effect: Std. Estimat*e* = 0.126). The mediating effect of entrapment and defeat accounted for 50.3% of the association between sleep disturbance and anxiety symptoms.

**Conclusion:**

Sleep disturbance and anxiety symptoms were prevalent among asymptomatic COVID-19 carriers. Entrapment and defeat mediate the association between sleep disturbance and anxiety symptoms. More attention is needed to monitoring sleep conditions and feelings of defeat and entrapment to reduce the risk of anxiety.

## Highlights

A cross-sectional study was conducted among 1,283 asymptomatic COVID-19 carriers during the 2022 SARS-CoV-2 Omicron outbreak in Shanghai.

Asymptomatic COVID-19 carriers had a high risk of developing anxiety symptoms and sleep disturbance during the pandemic.

Feelings of entrapment and defeat mediated the association between sleep disturbance and anxiety symptoms.

## Introduction

Coronavirus disease-19 (COVID-19) is widespread worldwide and has been defined by the World Health Organization as a severe public health emergency. The COVID-19 outbreak poses a great threat to public health, bringing with it a considerable burden of mental health problems. During the COVID-19 pandemic, there has been a significant increase in global mental health disorders, with depression and anxiety increasing by 27.6% and 25.6% respectively [1]. In addition, mental health problems were even more severe among those infected with COVID-19 compared to the general population [2]. A previous meta-analysis suggests that coronavirus infection can lead to a range of psychiatric disorders such as delirium, anxiety, depression, and insomnia [3]. In a cohort study among 68 million individuals in the USA, survivors of the COVID-19 had a higher risk of developing psychiatric sequelae [4]. Another survey in China at the beginning of the pandemic revealed the psychological impacts of the outbreak, with 28.8% of the population reporting moderate to severe anxiety symptoms [5]. In contrast, the prevalence of anxiety symptoms was higher in COVID-19 patients [6].

Several studies have focused on the etiology and risk factors of anxiety symptoms in people with COVID-19, finding that gender, age, marital status, COVID-19 disease duration, family infection, oxygen saturation levels, etc. were associated with anxiety symptoms [7, 8]. However, to facilitate effective psychological interventions in this population, there is a need to explore modifiable risk factors of anxiety symptoms. Sleep disturbance, another mental health problem prevalent in the context of the pandemic, could play a significant role in the development of anxiety symptoms [9]. Previous studies have demonstrated that sleep disturbance is a critical influencing factor of anxiety symptoms in people with COVID-19 [10, 11].

To better understand the relationship between sleep disturbance and anxiety symptoms, previous studies have explored the mediators linking these two disorders. Possible mediators included stress [12], personal sleep debt and daytime sleepiness [13], and cognitive complaints [14]. The Social Rank Theory proposes low mood and submissive behavior as involuntary adaptive responses to adverse situations. If the situations remain unchanged or inescapable, the adaptive responses will become maladaptive and eventually lead to serious pathology [15]. Entrapment (feelings of failed social struggle) and defeat (inability to escape from a situation) represent the key maladaptive stress responses in the process [16], which may underly the relationship between unfavorable sleep disturbance and anxiety symptoms. First, perceptions of defeat and entrapment have been identified as important precursors of different types of psychiatric disorders [17]. Previous studies showed that entrapment and defeat are closely related to anxiety symptoms [18, 19]. Furthermore, according to the Cry of Pain model, disturbed sleep could act as a stressor, triggering feelings of entrapment and defeat [20]. Although the relationship among sleep disturbance, entrapment, defeat, and anxiety symptoms remains unclear, previous studies have investigated the mediating effects of entrapment and defeat on the associations between insomnia symptoms and suicidal ideation, a more severe type of mental disorder [21, 22]. Similarly, we hypothesized that sleep disturbance could also increase the risk of anxiety symptoms through the mediating effects of entrapment and defeat.

Compared to the general population, evidence is limited on the mental health problems of the people with COVID-19. To the best of our knowledge, no study has yet explored the psychological mechanisms underlying the development of anxiety symptoms in this population. Therefore, we conducted a cross-sectional survey of anxiety symptoms, sleep disturbance, defeat, and entrapment in a group of asymptomatic COVID-19 carriers during the 2022 SARS-CoV-2 Omicron outbreak in Shanghai, when the SARS-CoV-2 were highly infectious but caused significantly lower mortality than the first wave of the outbreak in 2020 [23]. The aims of this study were: (1) to investigate the prevalence of sleep disturbance and anxiety symptoms in asymptomatic COVID-19 carriers in Shanghai, China; (2) to explore the mediating relationship between sleep disturbance, anxiety symptoms, defeat, and entrapment.

## Methods

### Participants and procedure

The study was conducted from March to April during the 2022 SARS-CoV-2 Omicron outbreak in Shanghai. Participants were asymptomatic COVID-19 carriers enrolled from the Ruijin Jiahe Fangcang Shelter Hospital, Shanghai via convenient sampling. The inclusion criteria were as follows: (1) aged 18 years and above; (2) have been diagnosed as asymptomatic COVID-19 carriers, which was confirmed by the threshold Cycle (Ct) value obtained from the real-time reverse transcriptase polymerase chain reaction (RT-PCR) test (a Ct value < 35 is considered positive); (3) consent to participate in the study (4) able to use a smartphone to complete the questionnaire independently. Self-reported questionnaires were completed through the online survey platform “Questionnaire Star” and then distributed via Wechat.

### Measurements

#### Pittsburgh Sleep Quality Index (PSQI)

The PSQI is a 19-item self-rated scale that measures seven components of sleep quality over the past month: subjective sleep quality, sleep latency, sleep duration, habitual sleep efficiency, sleep disturbances, use of sleeping medication, and daytime dysfunction [24]. The summed component scores yield a global score ranging from 0 to 21, with higher scores indicating poorer sleep quality. According to the recommended cut-off in the original study, a global score > 5 represents poor sleep quality [24]. The use of the PSQI in the Chinese adult population to assess sleep quality has been validated by previous studies with acceptable psychometric robustness and validated factor structure [25, 26].

#### Entrapment Scale (ES)

The 16-item ES was designed by Gilbert and Allan to evaluate the extent an individual feels imprisoned or trapped by unbearable thoughts, feelings, or circumstances [15]. The response of each item was assessed on a 5-point Likert scale from 0 (“not at all like me”) to 4 (“extremely like me”). Total scores of the ES range from 0 to 64, and higher scores indicate greater feelings of entrapment. Previous studies have demonstrated that the Chinese version of the ES had good reliability with a one-dimensional structure among men who have sex with men [27] and a two-dimensional structure among migrant workers [28]. The scale has also exhibited good internal consistency among college students (Cronbach’s alpha = 0.93–0.94) [29]. Cronbach’s alpha value for the scale was 0.976 in the present study.

#### Defeat Scale (DS)

The 16-item DS was designed by Gilbert and Allan to measure the sense of failed struggle and low social rank [15]. The response of each item was assessed on a 5-point Likert scale from 0 (“never”) to 4 (“always”). Total scores of the DS range from 0 to 64, and higher scores indicate more easily feeling defeat in daily life. The Chinese version of the DS has exhibited good internal consistency among college students (Cronbach’s alpha = 0.933), preferring a two-dimensional structure including decadence and low sense of achievement [30]. Cronbach’s alpha value for the scale was 0.912 in the present study.

#### Self-rating Anxiety Scale (SAS)

The 20-item SAS is an instrument for assessing individuals’ subject feelings of anxiety responding [31]. The response of each item was rated on a 4-point Likert scale from 1 (“none or a little of the time”) to 4 (“most of the time”). Total raw scores of the SAS range from 20 to 80, with higher scores representing more severe anxiety symptoms. The standard scores are produced by the raw scores multiplied by 1.25 (range from 25 to 100). The presence of anxiety was defined as standard scores > 50 [31]. The Chinese version of the SAS is reliable and well-validated [32]. The scale has also exhibited good internal consistency among elderly caregivers and college students (Cronbach’s alpha = 0.820–0.896) [33, 34]. Cronbach’s alpha value for the scale was 0.821 in the present study.

#### Background characteristics

Background characteristics included gender (male; female), age (18 ~ 34 years, 35 ~ 49 years, 50 ~ 71 years), education level (junior high school and below, college and above), marital status (unmarried, married, divorced, or widowed), days since diagnosis (≤ 7 days, 8 ~ 14 days, ≥ 8 days), and whether they stayed with families (yes, no).

### Statistical analysis

Descriptive analyses were first conducted for sociodemographic characteristics and the prevalence of sleep quality and anxiety symptoms. As the distribution of SAS scores was skewed, rank-sum tests were used to compare the SAS scores across different groups. Participants were categorized into two groups based on SAS scores: Anxiety and non-anxiety. Univariable logistic regressions were then performed to examine the associations between background variables and anxiety symptoms. Moreover, pairwise correlation analyses of the measurements (PSQI for sleep quality, entrapment scale for entrapment, defeat scale for defeat, and SAS for anxiety symptoms) were conducted to investigate the relationship among these variables.

Finally, the hypothetic mediation model for sleep quality, entrapment, defeat, and anxiety symptoms was tested using Preacher and Hayes’s method [35]. Bootstrapping analysis with 5000 resamples was conducted to derive bias-corrected 95% confidence intervals (*CI*). The weighted least squares and mean and variance estimator was used as the outcome was categorical. The significant background variables of anxiety symptoms were controlled in the mediation model. Goodness-of-fit of the model was evaluated by a series of model fit indices (root mean square error of approximation [RMSEA], comparative fit index [CFI], Tucker–Lewis index [TLI], standardized root mean square residual [SRMR]). RMSEA < 0.08, CFI > 0.90, TLI > 0.90, and SRMR < 0.08 indicated acceptable goodness-of-fit. *P-values* < 0.05 were considered statistically significant. All statistical analyses were performed using Mplus.

### Ethic

This study protocol was approved by the Ethics Committee of Ruijin Hospital, Shanghai Jiao Tong University School of Medicine. All subjects consented to participate in this study and provided their written informed consent.

## Results

### Descriptive analyses

As shown in Table 1, the final sample consisted of 765 males (59.6%) and 518 females (40.4%), ranging in age from 18 to 71 years (mean age = 39.6 ± 11.1 years). Most participants (73.9%, *n* = 948) were married, and 70.6% (*n* = 906) had an educational level of junior high school and below. Since the diagnosis of COVID-19, approximately half (50.4%, *n* = 646) of the participants had passed 8 ~ 14 days, and 65.9% (*n* = 846) did not stay with their family members. The prevalence rates of anxiety symptoms and sleep disturbance were 18.8% (*n* = 241) and 34.3% (*n* = 440), respectively.

**Table 1:** Descriptive characteristics of asymptomatic COVID-19 carriers

	Total (*n* = 1283)	SAS score		Anxiety	
		*M (Q1-Q3)*	*p value*	*n (%)*	*OR (95%CI)*
**Gender**					
Male	765 (59.6%)	35 (29–37)	0.346	133 (55.2%)	Ref
Female	518 (40.4%)	35 (29–38)		108 (44.8%)	1.252 (0.944–1.661)
**Age**					
18 ~ 34 years	469 (36.6%)	35 (29–38)	0.132	100 (41.5%)	Ref
35 ~ 49 years	532 (41.5%)	34 (20–36)		92 (38.2%)	0.772 (0.563–1.057)
50 ~ 71 years	282 (22.0%)	35 (29–37)		49 (20.3%)	0.776 (0.531–1.134)
**Education level**					
Junior high school and below	906 (70.6%)	35 (30–37)	0.007**	161 (66.8%)	Ref
College and above	377 (29.4%)	32 (27–38)		80 (33.2%)	1.246 (0.923–1.682)
**Marital status**					
Unmarried	285 (22.2%)	35 (29–37.5)	0.913	59 (24.5%)	Ref
Married	948 (73.9%)	35 (29–37)		175 (72.6%)	0.867 (0.623–1.206)
Divorced or widowed	50 (3.9%)	34 (29–36.25)		7 (2.9%)	0.624 (0.267–1.457)
**Days since diagnosis**					
≤ 7 days	186 (14.5%)	35 (30–39)	0.153	42 (17.4%)	Ref
8 ~ 14 days	646 (50.4%)	35 (29–37)		118 (49.0%)	0.766 (0.515–1.140)
≥ 15 days	451 (35.2%)	34 (28–37)		81 (33.6%)	0.751 (0.493–1.142)
**Stay with family**					
Yes	437 (34.1%)	34 (29–36)	0.035*	68 (28.2%)	Ref
No	846 (65.9%)	35 (29–38)		173 (71.8%)	1.395 (1.025–1.898)*
**Sleep disturbance**					
Good sleeper	843 (65.7%)	33 (27–35)	< .001***	84 (34.9%)	Ref
Poor sleeper	440 (34.3%)	36 (32–44)		157 (65.1%)	5.013 (3.721–6.753)
**Anxiety symptoms**					
Non-anxiety	1042 (81.2%)	-	-	-	-
Anxiety	241 (18.8%)	-		-	-

*M* median, *Q1* First Quartile, *Q3* Third Quartile, *OR* Odds ratio, *CI* Confidence interval

^*^ p < 0.05

^**^ p < 0.01

^***^ p < 0.001

Of these sociodemographic factors, education level (*p* = 0.007) and whether the participants stayed with their families (*p* = 0.035) were significantly associated with anxiety symptoms. In addition, poor sleepers had a higher risk of developing anxiety symptoms compared with good sleepers (*OR [95%CI]* = 5.013 [3.721–6.753]).

### Correlation analyses

The median and upper and lower quartiles of the PSQI score, ES score, DS score, and SAS score were 4 (2–7), 17 (16–26), 28 (24–35), and 35 (29–37), respectively. The results of correlation analyses were presented in Table 2. There were positive and significant correlations among sleep disturbance, entrapment, defeat, and anxiety symptoms (*p* < 0.001).

**Table 2:** Descriptive characteristics of questionnaire scores and pairwise correlation analysis

	PSQI score	Entrapment scale score	Defeat scale score	SAS score
PSQI score	1			
Entrapment scale score	0.592***	1		
Defeat scale score	0.403***	0.646***	1	
SAS score	0.358***	0.436***	0.554***	1
*M (Q1-Q3)*	4 (2–7)	17 (16–26)	28 (24–35)	35 (29–37)

*PSQI* Pittsburgh sleep quality index, *SAS* Self-rating anxiety scale *M* median, *Q1* First Quartile, *Q3* Third Quartile

^***^ p < 0.001

### Mediation analyses

**Fig. 1:**
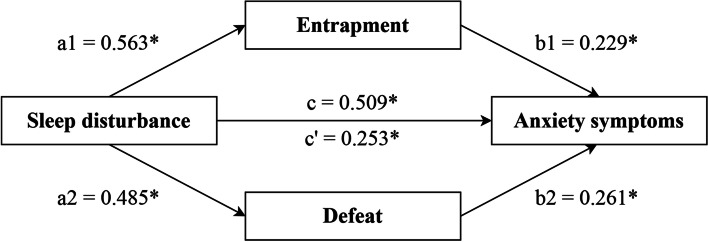
Feelings of entrapment and defeat mediate the association between sleep disturbance and anxiety symptoms ∗ *p* < 0.05. After adjusting for education level and whether the participants stayed with their families

As shown in Table 3 and Fig. 1, there was a significant total effect of sleep disturbance on anxiety symptoms (Standardized estimate [Std. estimate] = 0.509, p < 0.001) after adjusting for education level and whether the participants stayed with their families. When mediated through entrapment and defeat, the indirect effect of sleep disturbance on anxiety symptoms remained significant (Std. estimate = 0.253, *p* < 0.001). In addition, the indirect effect of sleep disturbance on anxiety symptoms through entrapment (Std. estimate = 0.129, p = 0.001) and defeat (Std. estimate = 0.126, p < 0.001) were both significant. Overall, the mediating effect of entrapment and defeat accounted for 50.3% (0.256/0.509 [Std. estimate of indirect effect/Std. estimate of total effect]) of the association between sleep disturbance and anxiety symptoms.

**Table 3:** Results of mediation analysis

Path	Std. Estimate	*S.E*	Est.*/S.E*	Bootstrapping *95% CI*		*P Valu*e
				**Lower 2.5%**	**Upper 2.5%**	
**Coefficient**						
Sleep disturbance → Anxiety	0.253	0.033	7.607	0.188	0.319	< .001
Sleep disturbance → Entrapment	0.563	0.023	24.151	0.516	0.607	< .001
Sleep disturbance → Defeat	0.485	0.025	19.448	0.435	0.533	< .001
Entrapment → Anxiety	0.229	0.043	5.332	0.142	0.313	< .001
Defeat → Anxiety	0.261	0.043	6.081	0.176	0.344	< .001
**Effect**						
**Total effect**	0.509	0.029	17.586	0.452	0.565	< .001
**Direct effect**						
Sleep disturbance → Anxiety	0.253	0.033	7.607	0.188	0.319	< .001
**Indirect effect**						
**Total indirect effect**	0.256	0.021	12.340	0.215	0.298	< .001
**Specific indirect effect**						
Sleep disturbance → Entrapment → Anxiety	0.129	0.025	5.072	0.080	0.180	< .001
Sleep disturbance → Defeat → Anxiety	0.126	0.022	5.838	0.085	0.170	< .001

Std. Estimate: Standardized estimate, *S.E* Standard error, *CI* Confidence interval

After adjusting for education level and whether the participants stayed with their families

The proposed mediation model showed acceptable goodness of fit (CFI = 0.991, TLI = 0.973, RMSEA = 0.058, SRMR = 0.069). All path coefficients in the model had *p values* < 0.05.

## Discussion

This study aimed to investigate the prevalence of sleep disturbance and anxiety symptoms among asymptomatic COVID-19 carriers in Shanghai, China, as well as to elucidate the role of entrapment and defeat underlying the association between these two psychological disorders. In this study, the prevalence rates of sleep disturbance and anxiety symptoms were 34.3% and 18.8%, respectively. Sleep disturbance could increase the risk of anxiety symptoms through the mediating effects of entrapment and defeat.

A total of 18.8% of the asymptomatic COVID-19 carriers experienced anxiety symptoms in the present study, which is similar to the prevalence rates (16.6%) previously reported in COVID-19 patients and healthcare workers who had a history of exposure [36, 37]. In contrast, moderate-to-severe anxiety was significantly less prevalent in the general population in China during the pandemic [38]. Although the severity of psychological problems varied across different stages of the pandemic, people with COVID are consistently vulnerable to anxiety symptoms. The high risk of anxiety symptoms in asymptomatic carriers, despite the absence of physical disorder, might be attributed to their fear of illness, experience of being stigmatized, and social isolation during quarantine [39]. A previous longitudinal study has shown that social isolation was among the determinants of widespread stress during the pandemic, which in turn resulted in impaired mental health [40].

Moreover, the prevalence of anxiety symptoms (35.7%) was even higher in the poor sleeper group. Compared to the asymptomatic carriers with normal sleep quality, the individuals with sleep disturbance had a higher risk of developing anxiety symptoms (*OR [95%CI]* = 5.013 (3.721–6.753)). This is consistent with previous findings that sleep disturbance is positively associated with the severity of anxiety symptoms during the pandemic [9]. Sleep disturbance could increase the risk of anxiety through a shared and mutually reinforcing neurocircuitry involving dopamine, serotonin, and adenosine [41]. Additionally, sleep could act as a moderator in the relationship between external stress and anxiety symptoms [37]. Therefore, as a modifiable factor, sleep conditions have significant potential in reducing anxiety symptoms, especially among asymptomatic COVID-19 carriers who are exposed to a number of psychosocial risk factors [42].

We found that participants with an education level of college and above reported a lower level of anxiety symptoms (*p* = 0.007). This is similar to previous findings that education attainment plays a protective role in mental health during the COVID-19 pandemic [43]. Our result also indicated that individuals who stayed with their families after being diagnosed with COVID-19 had a lower risk of developing anxiety symptoms (*OR [95%CI]* = 1.395 [1.025–1.898]). In contrast, those without family companions have reduced social interactions and are more likely to feel helpless in response to the pandemic. Several previous studies have revealed that living alone during the pandemic is a significant risk factor for anxiety symptoms in the adult population [44, 45]. The present study further demonstrated the need for family support among asymptomatic COVID-19 carriers to prevent anxiety symptoms.

After adjusting for education level and whether they stayed with families, feelings of entrapment and defeat mediated the association between sleep disturbance and anxiety symptoms in asymptomatic COVID-19 carriers. Although there is evidence regarding the mediating role of entrapment and defeat in the relationship between sleep disturbance and suicidal ideation [21, 22], no study has examined this psychological mechanism for less severe but more prevalent anxiety symptoms. Our results showed significant mediating effects of entrapment (25.3%) and defeat (24.8%) between the association of sleep disturbance and anxiety symptoms. Although the nature of the association of sleep disturbance with defeat and entrapment remains unclear, individuals with disturbed sleep may tend to experience daily difficulties with cognitive, emotional, interpersonal, and physical functioning, resulting in poorer quality of life and stronger feelings of being defeated [21]. Meanwhile, the difficulties in initiating or maintaining sleep block the escape route from daily problems through sleep, causing poor sleepers to feel trapped by their own thoughts or external environment [46]. The feelings of entrapment and defeat could subsequently increase the risks of anxiety symptoms [47].

Currently, studies of the mediation model of the associations between sleep disturbance, entrapment, defeat, and anxiety symptoms are new in the context of the COVID-19 pandemic. To the best of our knowledge, there is no study to examine the psychological mechanism underlying the development of anxiety symptoms in asymptomatic COVID-19 carriers, a vulnerable group at risk of psychological disorders during the pandemic. Our study identified sleep disturbance as a modifiable risk factor that could affect anxiety symptoms by increasing the feelings of defeat and entrapment. Therefore, interventions are needed for the asymptomatic carriers experiencing sleep disturbance. For example, cognitive behavioral therapy for insomnia (CBTi), a psychological treatment that can be delivered in non-clinical environment [48], can be used to reduce the potential risk of anxiety symptoms among poor sleepers. In addition, considering the mediating effects of entrapment and defeat in the relationship between sleep disturbance and anxiety symptoms, it’s necessary to monitor the levels of defeat and entrapment among those with sleep disturbance. Early identification and relief of defeat and entrapment may effectively block the effects of sleep disturbance on anxiety symptoms. Psychological counseling and support can benefit from boosting coping strategies that address the feelings of difficulties and frustration, preventing long-term mental health outcomes in poor sleepers [49].

The present study has several limitations. First, we should be cautious in drawing causal conclusions due to the cross-sectional study design. Longitudinal studies are needed to establish the temporal relationship between sleep disturbance, entrapment, defeat, and anxiety symptoms. Second, we relied on self-reported measures, which may be affected by recalling bias or socially desirable responses. However, given the sample size and the research setting of the present study, it’s acceptable to use the validated and frequently used self-reported scale (i.g. PSQI, ES, DS, and SAS) to measure the studied variables. In addition, we ensured information confidentiality to maximize the reliability and accuracy of the responses as possible. Third, in addition to the two mediators investigated in this study, other factors may also be critical in linking sleep disturbance to anxiety symptoms. For example, resilience was found to mediate the association between sleep disturbance and mental health problems [50]. Further exploration of multiple mediating effects could contribute to a better understanding of the pathway.

## Conclusions

This study is the first to explore the relationship among sleep disturbance, entrapment, defeat, and anxiety symptoms in asymptomatic COVID-19 carriers. In the present study, sleep disturbance and anxiety symptoms were prevalent among asymptomatic COVID-19 carriers. We also found that entrapment and defeat mediate the association between sleep disturbance and anxiety symptoms. We recommend that monitoring of sleep conditions and feelings of defeat and entrapment be strengthened to prevent anxiety in vulnerable populations.

## Data Availability

The datasets generated and/or analysed during the current study are not publicly available due to privacy or ethical restrictions but are available from the corresponding author on reasonable request.

## Abbreviations

COVID-19: Coronavirus disease-19
PSQI: Pittsburgh Sleep Quality Index
ES: Entrapment Scale
DS: Defeat Scale
SAS: Self-rating Anxiety Scale
RMSEA: Root mean square error of approximation
CFI: Comparative fit index
TLI: Tucker–Lewis index
SRMR: Standardized root mean square residual
