# Correction to: sleep disturbance and anxiety symptoms among asymptomatic COVID-19 carriers in Shanghai, China: the mediating role of entrapment and defeat

**DOI:** 10.1186/s12889-023-16064-1

**Published:** 2023-06-15

**Authors:** Yujie Liu, Xin Ge, Jinxin Zhang, Lulu Xu, Fan Hu, Suping Wang, Jialin Liu, Xiaodong Yang, Dake Shi, Yong Cai

**Affiliations:** 1 Tongren Hospital, Hongqiao International Institute of Medicine, Shanghai Jiao Tong University School of Medicine, Shanghai, 200335 China; 2 School of Public Health, Shanghai Jiao Tong University School of Medicine, Shanghai, 200025 China; 3 Department of Critical Care Medicine, Ruijin Hospital, Shanghai Jiao Tong University School of Medicine, Shanghai, 200025 China; 4 Department of Neurology, Institute of Neurology, Ruijin Hospital, Shanghai Jiao Tong University School of Medicine, Shanghai, 200025 China; 5 Department of Infection Control, Ruijin Hospital, Shanghai Jiao Tong University School of Medicine, Shanghai, 200025 China

**Correction to: BMC Public Health (2023) 23:993**

10.1186/s12889-023-15803-8

The original publication of this article contained an incorrect funding section. The incorrect and correct information is listed in this correction article, the original publication has been updated.

Incorrect

This work was support by Science and Technology Commission Shanghai Municipality (No. 20JC1410204) for the Seroepidemiological Study of Novel Coronavirus Pneumonia in Key Populations.

Correct

This work was support by the Shanghai Municipal Health Commission [grant numbers GWV-10.1-XK18] and Science and Technology Commission Shanghai Municipality (No. 20JC1410204) for the Seroepidemiological Study of Novel Coronavirus Pneumonia in Key Populations.

